# Convergence of Crisis: A Case Report of Diabetic Ketoacidosis Masking an Impending Thyroid Storm and Periodic Paralysis

**DOI:** 10.7759/cureus.61628

**Published:** 2024-06-03

**Authors:** Abizar Rangoonwala, Abhishek Sinha, Aditya Jain, Ahmed Afsa

**Affiliations:** 1 Emergency Medicine, United Lincolnshire Hospitals NHS trust, Boston, GBR; 2 Emergency Medicine, United Lincolnshire Hospitals NHS Trust, Boston, GBR

**Keywords:** proximal muscle wekness, hyperthyroidism, type 1 diabetes mellitus (t1d), hypokalaemic periodic paralysis, persistent hypokalemia, acute management of thyrotoxicosis, impending thyroid storm, diabetic ketoacidosis (dka)

## Abstract

Diabetic ketoacidosis (DKA) is an extreme complication of diabetes mellitus characterized by hyperglycemia, metabolic acidosis, and ketonemia. Thyroid storm, a potentially life-threatening manifestation of thyrotoxicosis, presents with a multitude of symptoms, including hyperthermia, tachycardia, and altered mental status. Periodic paralysis can be precipitated by different metabolic disturbances, including thyrotoxicosis, and may lead to extreme episodes of muscle weakness and paralysis. We present a case of a 41-year-old female with a history of type 1 diabetes mellitus and hyperthyroidism, who presented with DKA complicated by an impending thyroid storm and likely periodic paralysis exacerbated due to hypokalemia. Prompt recognition and aggressive management of each component of this triad were essential for a positive patient outcome.

This case highlights the importance of a broad and comprehensive approach to managing complex metabolic emergencies, particularly in patients with multiple comorbidities. Our patient presented to the emergency department with symptoms of severe vomiting, shortness of breath, and altered mental status. Laboratory investigations revealed metabolic derangements consistent with DKA, alongside impending thyrotoxicosis and hypokalemia-induced periodic paralysis. Management involved aggressive fluid resuscitation, insulin therapy, anti-thyroid medications, and potassium supplementation, with a multidisciplinary approach to stabilize the patient's condition.

## Introduction

This case highlights the complex correlation between endocrine and metabolic derangements in diabetes, thyroid dysfunction, and its neuromuscular presentations, presenting a diagnostic and therapeutic challenge. Diabetic ketoacidosis (DKA) is the most common hyperglycemic emergency presenting to a hospital. A diagnosis of DKA is confirmed when all three criteria are present: an abnormally elevated blood glucose level and/or a family history of diabetes mellitus; the presence of high blood and/or urine ketones; and metabolic acidosis with a high anion gap [1]. 

Thyroid storm is a severe form of thyrotoxicosis with symptoms such as high fever, rapid heart rate, and changes in mental status. The occurrence of thyroid storm is reported to be less than 10% in patients admitted for thyrotoxicosis, but the mortality rate is accounted to be 20-30% if this disease is not treated promptly [2]. 

Periodic paralyses are rare diseases characterized by severe episodes of muscle weakness due to changes in potassium levels in the blood. The disease is classified into hypokalemic, normokalemic, and hyperkalemic periodic paralysis. These are mainly due to a genetic etiology having an autosomal-dominant mode of inheritance, except for thyrotoxic hypokalemic periodic paralysis and periodic paralyses secondary to permanent changes in blood potassium levels [3].

## Case presentation

A 41-year-old female with a past medical history of type 1 diabetes mellitus, diabetic retinopathy, hyperthyroidism, hypertension, irritable bowel syndrome (IBS), and eczema presented to the emergency department with a three-day history of persistent vomiting, oral intolerance, generalized weakness, and dyspnea on minimal exertion. She was compliant with her diabetes medications. Additionally, she had been advised to stop her Carbimazole two years ago due to having euthyroid TFTs.

Upon examination, the patient was conscious, with a Glasgow Coma Scale (GCS) score of 15/15, a heart rate of 161 beats per minute, and a blood pressure of 140/100 mmHg. She was tachypneic with a temperature of 38°C and an oxygen saturation of 94%. Laboratory investigations showed a blood glucose level of 23 mmol/L and ketones measuring 6.4 mmol/L. Venous blood gas analysis was suggestive of severe metabolic acidosis (pH 7.17, HCO3 8.9), consistent with DKA. The patient appeared acutely unwell, pale, and extremely lethargic. Subsequent venous blood gas analyses showed gradual improvement in metabolic acidosis, but severe hypokalemia persisted (potassium: 2.5 mmol/L).

The DKA protocol involving fluid resuscitation, insulin therapy, and potassium supplementation was initiated; however, there was no significant improvement in the patient's clinical presentation. She still had persistent tachycardia despite the correction of volume status.

Given the persistent symptoms, an alternative etiology was considered. The Burch-Wartofsky Point Scale score was 50, indicative of an impending thyroid storm, and thyroid function tests showed severe hyperthyroidism (TSH <0.01, FT4 >100), supporting our suspicion. Subsequently, the patient was initiated on hydrocortisone, propylthiouracil, cholestyramine, and propranolol therapy.

The patient was transferred to the Intensive Treatment Unit (ITU) for further treatment on the same day, where she experienced an episode of muscle flaccid weakness within six hours of transfer. The episode lasted for approximately 48 hours. The weakness was more pronounced in the proximal muscles with reduced deep tendon reflexes. Compounding these findings with concurrent severe hypokalemia and muscle weakness resolving on potassium correction led to the diagnosis of periodic paralysis. 

**Table 1:** Burch-Wartofsky score for thyroid storm <25 points, unlikely to represent thyrotoxic crisis; 25-44 points, suggestive of impending crisis; ≥45 points, highly suggestive of thyrotoxic crisis - rapid, aggressive, specific, and supportive treatment required.

Criteria	Points
Temperature	
38-38.5 °C	5
38.6-39 °C	10
39.1-39.5 °C	15
39.6-40 °C	20
40.1-40.6 °C	25
>40.6 °C	30
Central nervous system disturbance	
Absent	0
Mild (agitation)	10
Moderate (delirium, psychosis, extreme lethargy)	20
Severe (seizure, coma)	30
Gastrointestinal-hepatic dysfunction	
Absent	0
Moderate (diarrhea, abdominal pain, nausea/vomiting)	10
Severe (jaundice)	20
Cardiovascular dysfunction	
Tachycardia (beats/min)	
<90/min	0
90-109/min	5
110-119/min	10
120-129/min	15
130-139/min	20
≥140/min	25
Congestive heart failure	
Absent	0
Mild (pedal oedema)	5
Moderate (bibasilar rales)	10
Severe (pulmonary edema)	15
Atrial fibrillation	
Absent	0
Present	10
A precipitating event	
Absent	0
Present	10

Diagnosis: DKA with concurrent impending thyroid storm and periodic paralysis 

Rationale and Differential Diagnoses

DKA: The patient presents with a known history of type 1 diabetes mellitus and exhibits classic symptoms of DKA, including vomiting, inability to tolerate oral intake, dyspnea on minimal exertion, and metabolic derangements such as hyperglycemia (blood glucose 23 mmol/L) and ketosis (ketones 6.4 mmol/L). Venous blood gas analysis confirms metabolic acidosis (pH 7.17, HCO3: 8.9 mmol/L), findings consistent with DKA. The initial treatment response to fluid resuscitation and insulin therapy is insufficient, indicating the need for further evaluation.

Impending thyroid storm: The patient demonstrates clinical signs and symptoms suggestive of an impending thyroid storm, including tachycardia (heart rate 161 bpm), hypertension (blood pressure 140/100 mmHg), palpitations, and a Burch-Wartofsky Point Scale score of more than 50. Thyroid function tests reveal hyperthyroidism, confirming the presence of thyrotoxicosis.

Periodic paralysis: The patient developed an episode of proximal muscle weakness and paralysis accompanied by hypokalemia, consistent with periodic paralysis.

In primary hypokalemic periodic paralysis, potassium levels are low only during attacks. Two forms of this condition have been recognized: (1) thyrotoxic periodic paralysis, which is associated with thyrotoxicosis, and (2) familial hypokalemic periodic paralysis, which is a genetic disorder of autosomal-dominant inheritance [3]. The extent of muscular depolarization caused by hypokalemia is related to the severity of muscle weakness [3].

The patient experienced periods of muscle weakness prior to this presentation. However, no identifiable common trigger for periodic paralysis has been found, as she did not seek medical attention for this weakness, leading to no records of potassium levels at the time of the weakness. This necessitates a comprehensive neurological evaluation to consider another possible cause.

Management

Intravenous (IV) fluids and insulin therapy were initiated to reverse the severe metabolic acidosis, hyperglycemia, and ketosis. Simultaneously, a diagnosis of an impending thyroid storm was made, evidenced by a high Burch-Wartofsky Point Scale (BWPS) score and deranged thyroid function tests (TFTs). To prevent the progression of the thyroid storm, propylthiouracil, intravenous hydrocortisone, and propranolol were administered to stop thyroid hormone production, reduce hormone release, and alleviate adrenergic symptoms. The patient developed periodic paralysis secondary to hypokalemia, necessitating continuous potassium replacement alongside regular electrolyte monitoring.

Episodes of thyrotoxic periodic paralysis occur only during uncontrolled hyperthyroidism. Hence, the management is based upon controlling the hyperthyroid state. Potassium chloride may be given to improve muscle strength and prevent new episodes [3].

A coordinated approach involving the medical, endocrine, and intensive care departments focused on correcting electrolyte imbalances, stabilizing thyroid function, and alleviating paralysis symptoms, ensuring optimal patient outcomes through careful monitoring and prompt interventions.

Patients can learn to decrease their number of bouts of periodic paralysis by having a balanced diet, such as avoiding carbohydrates in hypokalemic periodic paralysis. Regular exercise has also proven to be advantageous, and continuing to exercise may help abort impending episodes. Ingestion of potassium chloride salts may also be useful to prevent or abort these episodes [3]. Commonly, DKA occurs in patients with a history of diabetes; hence, this can be largely avoidable through early identification, and by patient and public education.

**Table 2:** Investigation results GFR: glomerular filtration rate, TSH, thyroid-stimulating hormone, BNP: B-type natriuretic peptide.

Investigation	Unit	Range	On presentation	Post-treatment
Sodium	mmol/L	133-146	132	142
Potassium	mmol/L	3.5-5.3	2.0	4.1
Bicarbonate	mmol/L	22-29	8.9	15.2
Urea	mmol/L	2.5-7.8	6.0	2.0
Creatinine	umol/L	45-84	54	46
GFR	mL/min	90-200	>90	>90
Serum glucose	mmol/L	3-6	23.2	12.1
Hemoglobin	g/dL	117-149	107	117
White blood cells	10^9/L	4.3-11.2	20.7	9.0
TSH	mU/L	0.27-4.5	<0.01	<0.01
T4	pmol/L	11-23	>100	87.2
pH		7.35-7.45	7.1	7.375
Lactate	mmol/L	0.5-2.2	5.8	1.4
Ketone	mmol/L	<0.6	6.4	<0.6
Magnesium	mmol/L	0.70-1.0	0.51	0.70
BNP	ng/L	0-300	4390	3479

Outcome

The patient had a total stay of eight days in the hospital, during which her symptoms resolved, and she was optimized for discharge. During the hospital stay, she was kept under close observation and monitored regularly. Subsequently, the patient was discharged with guidance for follow-up care from both the diabetic and endocrinology teams for ongoing review and monitoring.

## Discussion

This case highlights the complex correlation between metabolic derangements in diabetes, thyroid dysfunction, and its neuromuscular presentations, presenting a diagnostic and therapeutic challenge. The simultaneous occurrence of DKA, impending thyroid storm, and periodic paralysis required a nuanced approach to treatment, adjusting the metabolic abnormalities while avoiding exacerbation of thyrotoxicosis-induced complications.

In a patient with coexisting type 1 DM and hyperthyroidism, compensatory mechanisms of DKA cause cortisol and catecholamine release, which, when combined with rapid metabolic shifts, trigger a thyroid storm [4]. Another theory suggests that excess thyroid hormone causes an impairment of carbohydrate metabolism, leading to an increase in intestinal carbohydrate absorption and hepatic glycogen breakdown, aggravating insulin resistance, and triggering DKA, ultimately leading to thyroid storm [5]. Increased circulating thyroid hormone alters carbohydrate metabolism, affecting gluconeogenesis, glycogen synthesis, glucose oxidation, non-oxidative glucose metabolism, adipokine signaling, and lipid oxidation, creating a hyperglycemic state and increasing insulin resistance [5]. It has been reported that the diagnosis of thyroid storm is found late in patients with DKA due to suppression of fever [6-8] and relatively reduced thyroid hormone levels [9]. Patients with thyrotoxicosis show marked insulin resistance to an oral glucose tolerance test that significantly improves with the control of hyperthyroidism [10]. In hyperthyroidism, there is an increase in the glomerular filtration rate, which leads to increased excretion of insulin and hence decreased insulin levels, aggravating DKA [11]. Hence, multiple factors cause a concurrent DKA and thyroid storm and exacerbate each other.

The frequency of thyrotoxic periodic paralysis (TPP) is unknown. It is present in all populations but is found more frequently among Asians, with a male-to-female ratio of 9:1 [3]. Previous studies of TPP have suggested an incidence of 2% among Asians with hyperthyroidism compared to just 0.1 to 0.2% in non-Asians with hyperthyroidism [12]. TPP episodes have been linked with various precipitating factors like high glucose intake, physical exertion, and ketoacidosis, which are likely precipitating factors in our case. The exact mechanism of TPP is unknown, but it is believed to be due to a sudden and large volume of intracellular uptake of potassium into muscles, leading to hypokalemia. Thyroid hormones increase ATPase activity and can increase the number and sensitivity of beta-receptors, causing intracellular potassium influx [13]. It is notable that our patient was taken off Carbimazole due to her euthyroid TFT status, which could have been one of the key factors leading up to this case. A systematic review reported only 26 cases globally for the concurrent presentation of DKA with thyroid storm and a high mortality rate of 15%. Hence, this is indicative of a rare presentation but a condition for which physicians should have a low threshold of suspicion [14].

## Conclusions

This case report highlights the importance of recognizing and managing the complex interactions between endocrine disorders and their diverse clinical presentations. Patient involvement and compliance with lifestyle changes, medications, and closer monitoring are key for better management of their health. Closer monitoring of patients being discontinued from anti-thyroid therapy should be considered, especially in those with coexisting endocrine disorders.

Clinicians should maintain a high index of suspicion for concurrent metabolic and hormonal abnormalities in patients presenting with overlapping symptoms, enabling timely intervention to optimize patient outcomes.
